# Correction to: Long telomere inheritance through budding yeast sexual cycles

**DOI:** 10.1093/genetics/iyaf188

**Published:** 2025-10-21

This is a correction to: Vasilisa Sidarava, Sarah Mearns, David Lydall, Long telomere inheritance through budding yeast sexual cycles, *Genetics*, Volume 231, Issue 1, September 2025, iyaf129, https://doi.org/10.1093/genetics/iyaf129

The file Sidavaratableschanges.docx has been removed from the originally published Supplementary Data files, but is attached to this notice for record of changes to the article. These were intended during production to replace Tables 1, 2, and 3 in the body of the paper. As further corrections were needed, the emended tables are now as outlined below.

In Table 1 Figure references are corrected; one strain, DLY 12569, is added; DLY 12581 had been mis-labelled 12571.

Table 1 should read:

**Table 1.** Strains used in this study.

Strain	Background	Genotype	Origin	Experiment
DLY 3001	W303	*MATα ade2-1 trp1-1 can1-100 leu2-3,112 his3-11,15 ura3 GAL + psi+ ssd1-d2 RAD5*	Lydall lab collection	*WT* haploid parent in Fig. 4, Supplementary Fig. 2 (lane 2)
DLY 8460	W303	*MATa ade2-1 trp1-1 can1-100 leu2-3,112 his3-11,15 ura3 GAL + psi+ ssd1-d2 RAD5*	Lydall lab collection	WT haploid parent in Fig. 5A (lane 2), Fig. 5B (lane 1), Supplementary Fig. 3 (lane 3)
DLY 2264	W303	*DLY 8460 tel1::URA3*	Lydall lab collection	*tel1Δ* in Supplementary Fig. 3
DLY 4451	W303	*DLY 3001 rif1::URA3*	Lydall lab collection	*rif2Δ* in Fig. 2
DLY 4457	W303	*DLY 3001 mre11::URA3*	Lydall lab collection	*mre11Δ* in Fig. 2
DLY 4528	W303	*DLY 8460 nmd2::HIS3*	Lydall lab collection	*nmd2Δ* in Supplementary Fig. 3
BY 4741 *rif2Δ::KANMX*	S288C	*MATa ura3 leu2 his3 rif2::KANMX*	Peter Banks, Newcastle University	Used to knockout *RIF2* in DLY 8460
DLY 6884	W303	*DLY 8460 yku70::LEU2*	Lydall lab collection	*yku70Δ* in Supplementary Fig. 3
DLY 11741	W303	*DLY 8460 mre11::URA3*	Lydall lab collection	*mre11Δ* in Supplementary Fig. 3
DLY 12522	W303	*DLY 3001 pDL:URA3*	Lydall lab collection	*WT* in Fig. 2 (lane 2)
DLY 12525	W303	*DLY 8460 pDL:TRP1*	Lydall lab collection	*WT* in Fig. 2 (lanes 7–12)
DLY 12528	W303	*DLY 8460 yku70::LEU2 pDL:TRP1*	Lydall lab collection	*yku70Δ* in Fig. 2
DLY 12531	W303	*DLY 8460 rif2::HIS3 pDL:TRP1*	Lydall lab collection	*rif2Δ* in Fig. 2
DLY 12555	W303	*DLY 3001 rif1::KANMX*	This study	*rif1Δ* clone A in Fig. 3
DLY 12558	W303	*DLY 8460 rif2::KANMX*	This study	*rif2Δ* clone A in Fig. 3, *rif2Δ* haploid parent in Fig. 4
DLY 12559	W303	*DLY 8460 rif2::KANMX*	This study	*rif2Δ* clone B in Fig. 3
DLY 12564	W303	*DLY 3001 rif1::KANMX*	This study	*rif1Δ* clone B in Fig. 3
DLY 12568	W303	*DLY 3001 long VIR telomere*	This study	*WT* haploid parent in Fig. 5A (lane 3), Supplementary Fig. 3 (lanes 2, 7, 12, 17, 22, 27–29)
DLY 12569	W303	*DLY 8460 rif2::KANMX*	This study	*rif2Δ* in Supplementary Fig. 2 (lane 3)
DLY 12581	W303	*DLY 3001 long VIR telomere*	This study	*WT* in Fig. 5B (lane 2)

instead of:

**Table 1.** Strains used in this study.

Strain	Background	Genotype	Origin	Experiment
DLY 3001	W303	*MATα ade2-1 trp1-1 can1-100 leu2-3,112 his3-11,15 ura3 GAL + psi + ssd1-d2 RAD5*	Lydall lab collection	*WT* in Fig. 4
DLY 8460	W303	*MATa ade2-1 trp1-1 can1-100 leu2-3,112 his3-11,15 ura3 GAL + psi + ssd1-d2 RAD5*	Lydall lab collection	*WT* in Fig. 5a (lane 2), Fig. 5b (lane 1), Supplementary Fig. 2 (lane 3)
DLY 2264	W303	*DLY 8460 tel1::URA3*	Lydall lab collection	*tel1Δ* in Supplementary Fig. 2
DLY 4451	W303	*DLY 3001 rif1::URA3*	Lydall lab collection	*rif2Δ* in Fig. 2
DLY 4457	W303	*DLY 3001 mre11::URA3*	Lydall lab collection	*mre11Δ* in Fig. 2
DLY 4528	W303	*DLY 8460 nmd2::HIS3*	Lydall lab collection	*nmd2Δ* in Supplementary Fig. 2
BY 4741 *rif2Δ::KANMX*	S288C	*MATa ura3 leu2 his3 rif2::KANMX*	Peter Banks, Newcastle University	Used to knockout *RIF2* in DLY 8460
DLY 6884	W303	*DLY 8460 yku70::LEU2*	Lydall lab collection	*yku70Δ* in Supplementary Fig. 2
DLY 11741	W303	*DLY 8460 mre11::URA3*	Lydall lab collection	*mre11Δ* in Supplementary Fig. 2
DLY 12522	W303	*DLY 3001 pDL:URA3*	Lydall lab collection	*WT* in Fig. 2 (lane 2)
DLY 12525	W303	*DLY 8460 pDL:TRP1*	Lydall lab collection	*WT* in Fig. 2 (lanes 7–12)
DLY 12528	W303	*DLY 8460 yku70::LEU2 pDL:TRP1*	Lydall lab collection	*yku70Δ* in Fig. 2
DLY 12531	W303	*DLY 8460 rif2::HIS3 pDL:TRP1*	Lydall lab collection	*rif2Δ* in Fig. 2
DLY 12555	W303	*DLY 3001 rif1::KANMX*	This study	*rif1Δ* clone A in Fig. 3
DLY 12558	W303	*DLY 8460 rif2::KANMX*	This study	*rif2Δ* clone A in Fig. 3, *rif2Δ* in Fig. 4
DLY 12559	W303	*DLY 8460 rif2::KANMX*	This study	*rif2Δ* clone B in Fig. 3
DLY 12564	W303	*DLY 3001 rif1::KANMX*	This study	*rif1Δ* clone B in Fig. 3
DLY 12568	W303	*DLY 3001 long VIR telomere*	This study	*WT* in Fig. 5a (lane 3), Supplementary Fig. 2 (lanes 2, 7, 12, 17, 22, 27–29)
DLY 12571	W303	*DLY 3001 long VIR telomere*	This study	*WT* in Fig. 5b (lane 2)

In Table 2, Figure references are corrected. Table 2 should read:

**Table 2.** Cell division numbers.

Cell origin	In a colony	In a patch	In liquid culture	Reference
Auxotrophically selected diploids	25 (from 1 to 2.8*10^7^ cells)	4 (from 1.4*10^7^ to 1.6*10^8^ cells)	3 (from 5*10^7^ to 2.9*10^8^ cells)	P1 diploids in Fig. 2, Supplementary Fig. 1
All diploids from P2 and beyond	25 (as above)	4 (as above)	3 (as above)	Diploids in Fig. 2, Supplementary Fig. 1, and Supplementary Fig. 3 (except P1);
Haploid transformants	26 (1st G418 plate: from 1 to 4.6*10^7^ cells) and 23 (2nd G418 plate: from 1 to 6.8*10^6^ cells)	5 (from 6.8*10^6^ to 1.6*10^8^ cells)	3 (est. from data for haploids)	P1 haploids in Fig. 3
Haploids from P2 and beyond	26 (from 1 to 7.9*10^7^ cells)	3 (from 2.0*10^7^ to 15.3*10^7^ cells)	3 (from 5.1*10^7^ to 3.6*10^8^ cells)	Haploids in Fig. 3 (except P1)
Pulled zygote	25 (from 1 to 2.8*10^7^ cells)		4 (from 1.4*10^7^ to 1.8*10^8^ cells)	Diploids in Figs. 4 and 5, and Supplementary Fig. 2; P1 in Supplementary Fig. 3
Germinated spore	25 (from 1 to 2.4*10^7^ cells)		4 (from 1.2*10^7^ to 2.1*10^8^ cells)	Haploid progeny in Figs. 4 and 5, and Supplementary Fig. 2

instead of:

**Table 2.** Cell division numbers.

Cell origin	In a colony	In a patch	In liquid culture	References
Auxotrophically selected diploids	25 (from 1 to 2.8*10^7^ cells)	4 (from 1.4*10^7^ to 1.6*10^8^ cells)	3 (from 5*10^7^ to 2.9*10^8^cells)	P1 diploids in Figs. 2, S1
All diploids from P2 and beyond	25 (as above)	4 (as above)	3 (as above)	Diploids in Fig. 2, S1, and S2 (except P1);
Haploid transformants	26 (1st G418 plate: from 1 to 4.6*10^7^ cells) and 23 (2nd G418 plate: from 1 to 6.8*10^6^ cells)	5 (from 6.8*10^6^ to 1.6*10^8^ cells)	3 (est. from data for haploids)	P1 haploids in Fig. 3
Haploids from P2 and beyond	26 (from 1 to 7.9*10^7^ cells)	3 (from 2.0*10^7^ to 15.3*10^7^ cells)	3 (from 5.1*10^7^ to 3.6*10^8^ cells)	Haploids in Fig. 3 (except P1)
Pulled zygote	25 (from 1 to 2.8*10^7^ cells)		4 (from 1.4*10^7^ to 1.8*10^8^ cells)	Diploids in Figs. 4 and 5; P1 in Supplementary Fig. 2
Germinated spore	25 (from 1 to 2.4*10^7^ cells)		4 (from 1.2*10^7^ to 2.1*10^8^ cells)	Haploid progeny in Figs. 4 and 5

In Table 3, The sequences recognized by M4813 and M4814 are corrected from *RIF2* to *RIF1*. The sequence of M4814 is corrected. Details of primers M4815 and M4816 have been added. Table 3 should read:

**Table 3.** Primers used in this study.

Primer	Annealing region	Sequence	Used for	Origin
M294	Y’-TG	CCCAGTCACGACGTTGTAAAACG	Y’-TG probe	M13/pUC Forward
M295	Y’-TG	AGCGGATAACAATTTCACACAGG	Y’-TG probe	M13/pUC Reverse
M1105	TEL06R	TAAAGGAATCCCCAGAGACCTC	VIR probe	(Zubko and Lydall 2006)
M1106	TEL06R	TTGCCACGCAAAGAAAGG	VIR probe	(Zubko and Lydall 2006)
M4813	*RIF1* knockout (S288C)	CGCTATCAGCCAAGTATAACG	Amplifying G418^r^ cassette inserted into *RIF1* locus of S288C strain to knock out *RIF1* in W303 background	This study
M4814	*RIF1* knockout (S288C)	CACAAATTAGAGTAAAACCCGACC	Amplifying G418 construct inserted into *RIF1* locus of S288C strain to knock out *RIF1* in W303 background	This study
M4815	*RIF2* knockout (S288C)	GAGCGTTGGAAATGATAGCG	Amplifying G418^r^ cassette inserted into *RIF2* locus of S288C strain to knock out *RIF2* in W303 background	This study
M4816	*RIF2* knockout (S288C)	GGTAAAGATCAGTCCATTCGG	Amplifying G418^r^ cassette inserted into *RIF2* locus of S288C strain to knock out *RIF2* in W303 background	This study
M4821	TEL03L	AGCTTTCATCATTCGCGCTGA	IIIL probe	Raymund Wellinger, pers. comm. (2024)
M4822	TEL03L	CGTCAACAGGTTATGAGCCCT	IIIL probe	Raymund Wellinger, pers. comm. (2024)
M4825	TEL15L	CTTCGTCAAAGGGATGAAAGC	XVL probe	This study
M4826	TEL15L	GGTAATGATGCTATGCTCTTAACG	XVL probe	This study

instead of:

**Table 3.** Primers used in this study.

Primer	Annealing region	Sequence	Used for	Origin
M294	Y’-TG	CCCAGTCACGACGTTGTAAAACG	Y’-TG probe	M13/pUC Forward
M295	Y’-TG	AGCGGATAACAATTTCACACAGG	Y’-TG probe	M13/pUC Reverse
M1105	TEL06R	TAAAGGAATCCCCAGAGACCTC	VIR Probe	(Zubko and Lydall 2006)
M1106	TEL06R	TTGCCACGCAAAGAAAGG	VIR Probe	(Zubko and Lydall 2006)
M4813	*RIF2* knockout (S288C)	CGCTATCAGCCAAGTATAACG	Amplifying G418^r^ cassette inserted into *RIF1* locus of S288C strain to knock out *RIF1* in W303 background	This study
M4814	*RIF2* knockout (S288C)	CGCTATCAGCCAAGTATAACG	Amplifying G418 construct inserted into *RIF1* locus of S288C strain to knock out *RIF1* in W303 background	This study
M4821	TEL03L	AGCTTTCATCATTCGCGCTGA	IIIL Probe	Raymund Wellinger, pers. comm. (2024)
M4822	TEL03L	CGTCAACAGGTTATGAGCCCT	IIIL Probe	Raymund Wellinger, pers. comm. (2024)
M4825	TEL15L	CTTCGTCAAAGGGATGAAAGC	XVL Probe	This study
M4826	TEL15L	GGTAATGATGCTATGCTCTTAACG	XVL Probe	This study

## Supplementary Material

iyaf188_Supplementary_Data

