# Supplementary material for: Long telomere inheritance through budding yeast sexual cycles
Source: Genetics. 2025 Jul 14;231(1):iyaf129. doi: 10.1093/genetics/iyaf129 (PMC12406008; doi:10.1093/genetics/iyaf129)
Supplement: iyaf129_Supplementary_Data [file iyaf129_supplementary_data.zip › Figure_S3C_GENETICS-2025-308237.pdf]

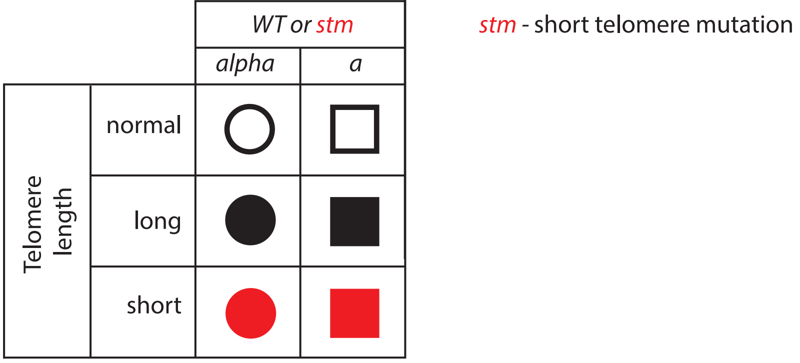
WT or stm
stm - short telomere mutation
alpha
a
Telomere length
normal
long
short

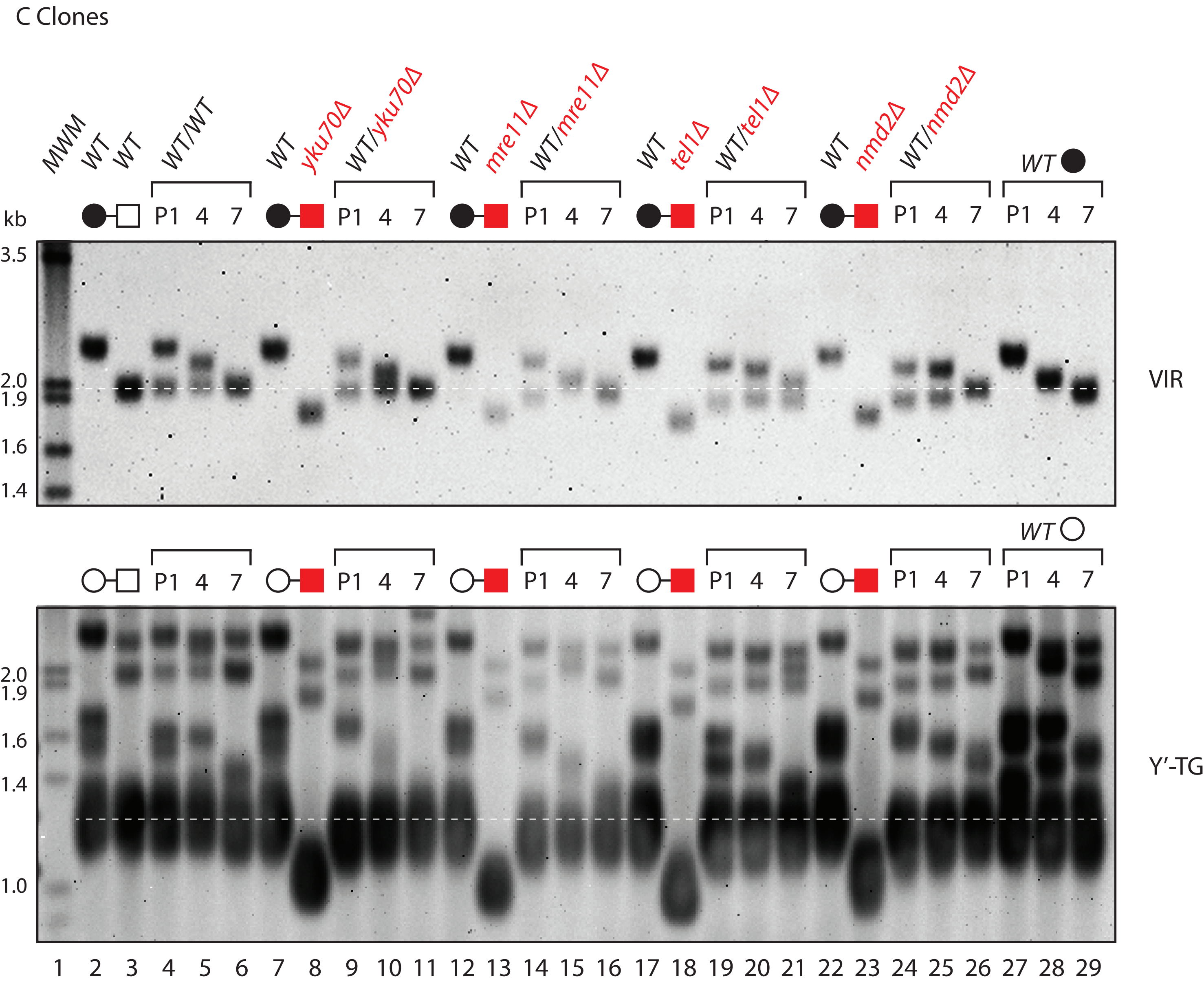
C Clones
MWM
WT
WT
WT/WT
WT
yku70Δ
WT/yku70Δ
WT
mre11Δ
WT/mre11Δ
WT
tel1Δ
WT/tel1Δ
WT
nmd2Δ
WT/nmd2Δ
WT
kb
P1 4 7
3.5
2.0
1.9
1.6
1.4
VIR
WT
Y'-TG
1.0
1 2 3 4 5 6 7 8 9 10 11 12 13 14 15 16 17 18 19 20 21 22 23 24 25 26 27 28 29

Figure S3C
