# Supplementary material for: Long telomere inheritance through budding yeast sexual cycles
Source: Genetics. 2025 Jul 14;231(1):iyaf129. doi: 10.1093/genetics/iyaf129 (PMC12406008; doi:10.1093/genetics/iyaf129)
Supplement: iyaf129_Supplementary_Data [file iyaf129_supplementary_data.zip › Supplemental_Material_Legends_GENETICS-2025-308237.docx]

**Supplemental Material legends**

**Figure S1.** *Set of diploid yeast produced by mating haploids with (WT), long (rif1∆, rif2∆) or short (mre11∆, yku70∆) telomeres as described in Figure 2.* Telomeres of diploids at passage 1 (32 divisions), 5 (132 divisions) and 9 (232 divisions) were detected by Southern blot using Y’-TG and VIR probes. Red stars indicate poorly digested samples. White lines indicate normal (wild-type) telomere length.

**Figure S2.** *A cross between WT and rif2∆ strains.* WT cells and *rif2∆* cells with long telomeres (from Figure 4, clone 3c) were mated and sporulated. Telomeres were detected using Y’-TG and VIR probes. Dashed white lines indicate normal (wild-type) telomere length.

**Figure S3.** *Telomere blots showing raw data used in Figure 6.* WT cells with long telomeres (from Figure 4 clone 3a) were mated *yku70∆, mre11∆, tel1∆ and nmd2∆* haploids. Four diploid clones (A–D) from each mating were passaged and examined by Southern blot. Telomeres at passage 1 (32 divisions), 4 (107 divisions) and 7 (182 divisions) were detected using Y’-TG and VIR probes. Dashed white lines indicate normal (wild-type) telomere length. A single haploid wild type clone, with a long VIR telomere was used as a control on all four blots (lanes 27–29).

**File S1.** *Estimations of telomere lengths for Figures 2–5, S1–S2*

**File S2.** *Analysis of segregation patterns of rif2∆, mating type and telomere lengths of VIR, XVL, and IIIL for Figures 4 and S2.*

**File S3.** *Estimations of VIR telomere lengths and VIR shortening rates for Figure S3.*
