# Supplementary figures and images for: Long telomere inheritance through budding yeast sexual cycles
Source: Genetics. 2025 Jul 14;231(1):iyaf129. doi: 10.1093/genetics/iyaf129 (PMC12406008; doi:10.1093/genetics/iyaf129)

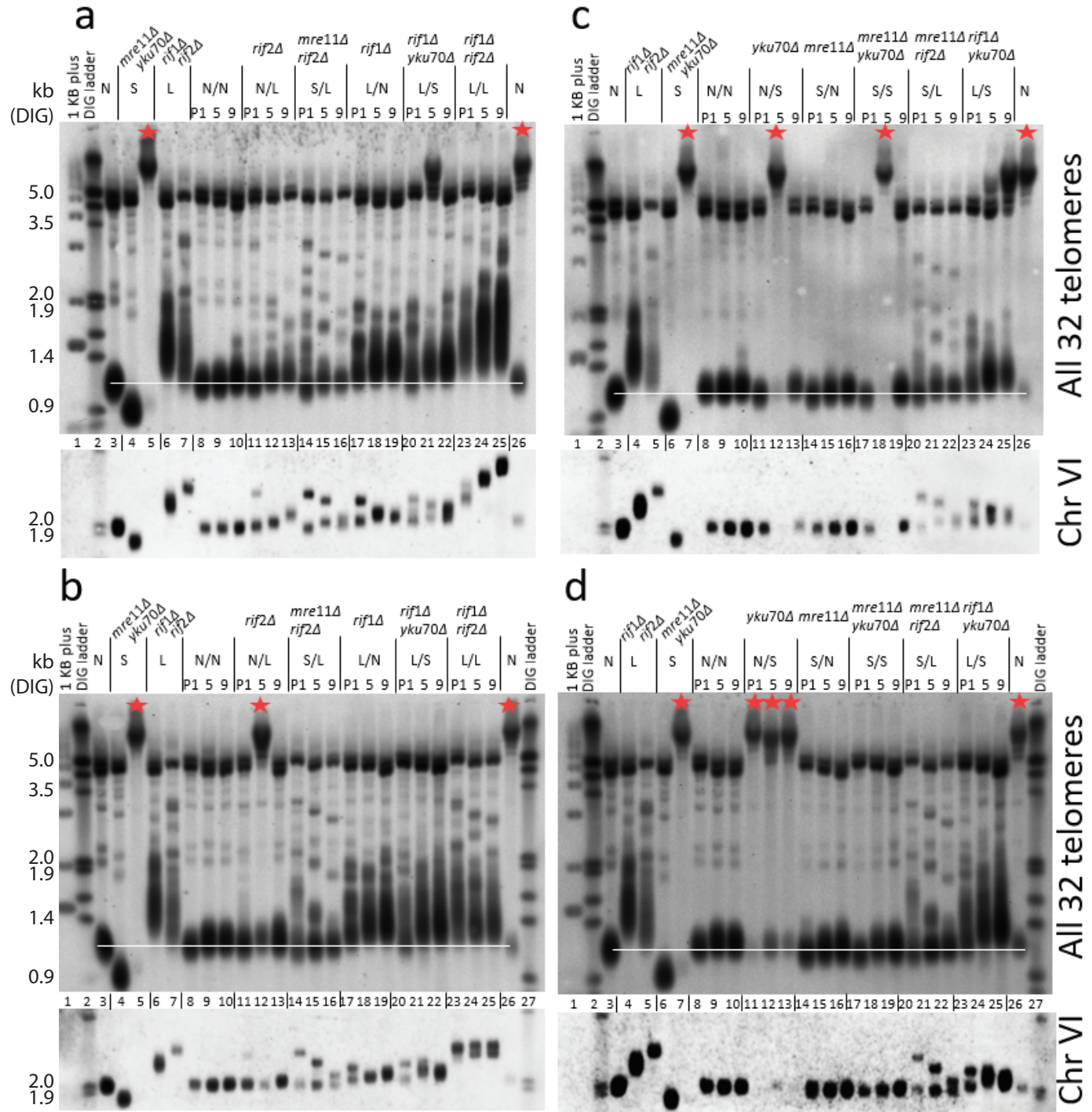

**Figure S1**

Supplement: iyaf129_Supplementary_Data [file iyaf129_supplementary_data.zip › Figure_S1_GENETICS-2025-308237.pdf]

|                 |        | WT                                                                                |                                                                                   | <i>rif2Δ</i>                                                                      |                                                                                   |
|-----------------|--------|-----------------------------------------------------------------------------------|-----------------------------------------------------------------------------------|-----------------------------------------------------------------------------------|-----------------------------------------------------------------------------------|
|                 |        | <i>alpha</i>                                                                      | <i>a</i>                                                                          | <i>alpha</i>                                                                      | <i>a</i>                                                                          |
| Telomere length | normal | 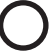 | 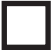 | 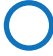 | 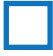 |
|                 | long   | 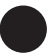 | 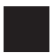 | 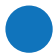 | 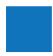 |

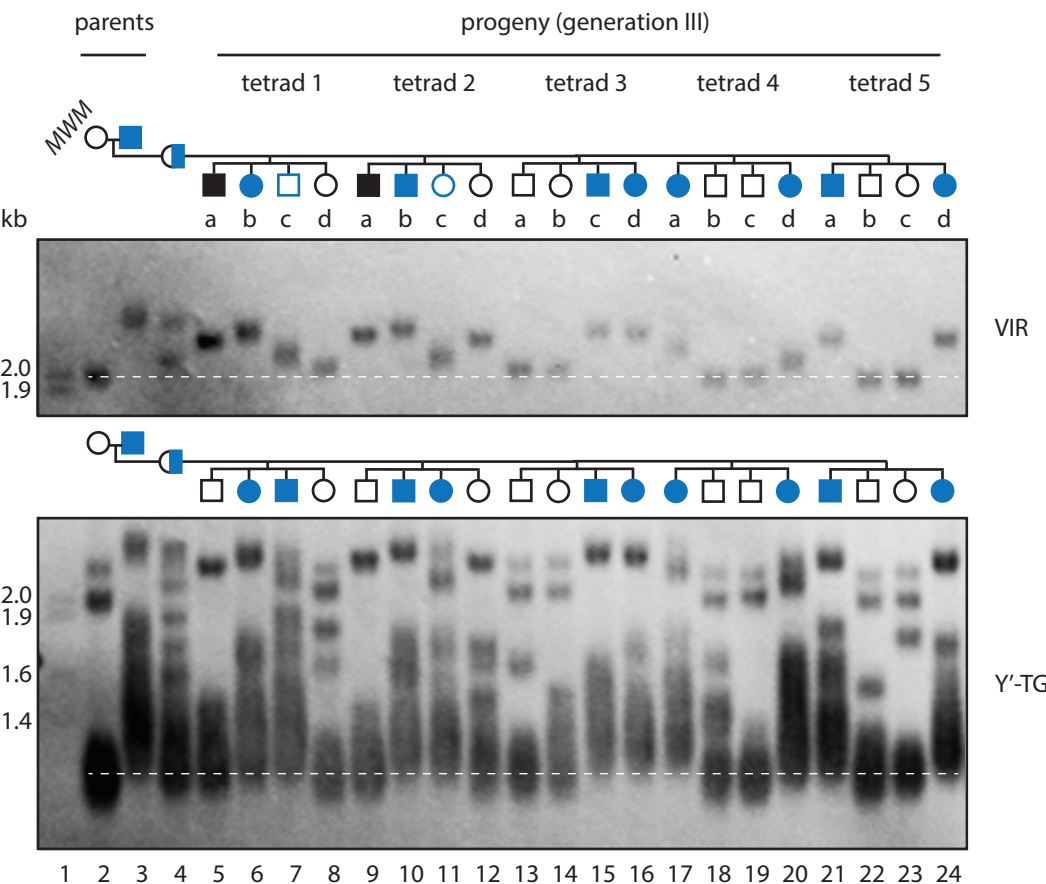

**Figure S2**

Supplement: iyaf129_Supplementary_Data [file iyaf129_supplementary_data.zip › Figure_S2_GENETICS-2025-308237.pdf]
