# Supplementary material for: Long telomere inheritance through budding yeast sexual cycles
Source: Genetics. 2025 Jul 14;231(1):iyaf129. doi: 10.1093/genetics/iyaf129 (PMC12406008; doi:10.1093/genetics/iyaf129)
Supplement: iyaf129_Supplementary_Data [file Figure_S3D_GENETICS-2025-308237.pdf › Figure_S3D_GENETICS-2025-308237.pdf]

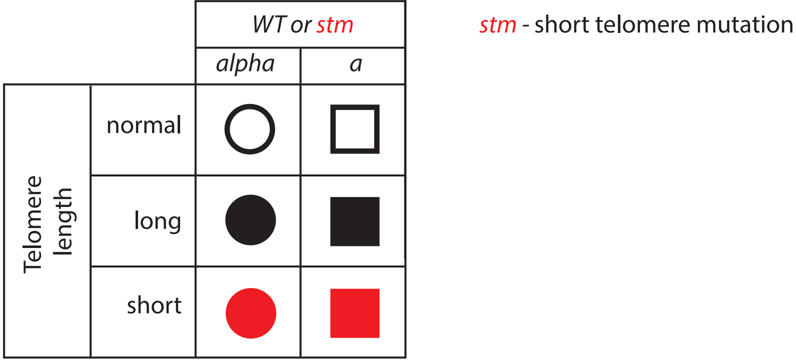

D Clones

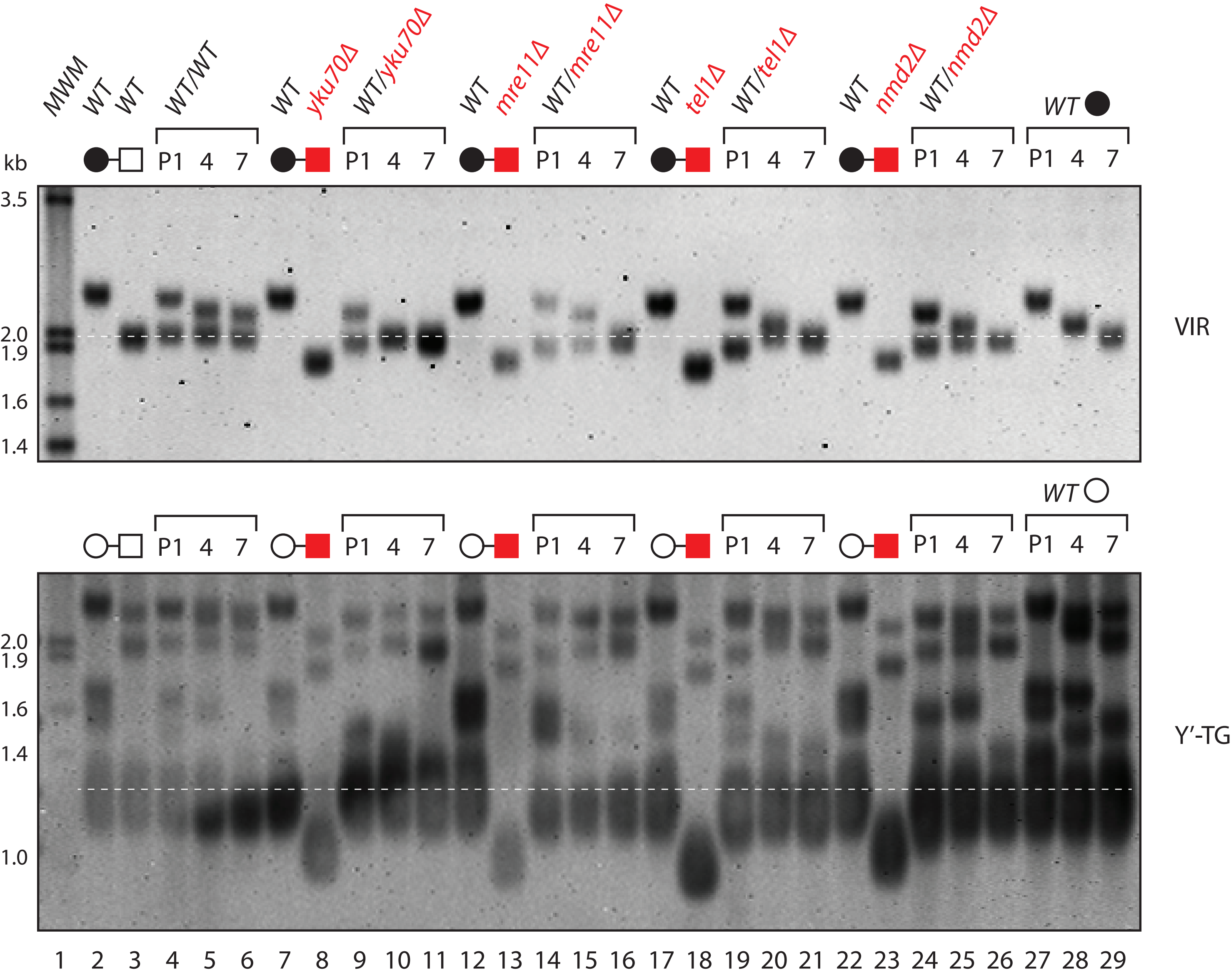

Figure S3D
