# Supplementary material for: Correction to: Long telomere inheritance through budding yeast sexual cycles
Source: Genetics. 2025 Oct 21;231(4):iyaf188. doi: 10.1093/genetics/iyaf188 (PMC12693589; doi:10.1093/genetics/iyaf188)
Supplement: iyaf188_Supplementary_Data [file iyaf188_supplementary_data.docx]

**Table 1.** Strains used in this study

| **Strain** | **Background** | **Genotype** | **Origin** | **Experiment** |
| --- | --- | --- | --- | --- |
| DLY 3001 | W303 | *MATα ade2-1 trp1-1 can1-100 leu2-3,112 his3-11,15 ura3 GAL+ psi+ ssd1-d2 RAD5* | Lydall lab collection | *WT* haploid parent in Figure 4, S2 (lane 2) |
| DLY 8460 | W303 | *MATa ade2-1 trp1-1 can1-100 leu2-3,112 his3-11,15 ura3 GAL+ psi+ ssd1-d2 RAD5* | Lydall lab collection | *WT* in Figure 5A (lane 2), Figure 5B (lane 1), Figure S3 (lane 3) |
| DLY 2264 | W303 | *DLY 8460 tel1::URA3* | Lydall lab collection | *tel1∆* in Figure S3 |
| DLY 4451 | W303 | *DLY 3001 rif1::URA3* | Lydall lab collection | *rif2∆* in Figure 2 |
| DLY 4457 | W303 | *DLY 3001 mre11::URA3* | Lydall lab collection | *mre11∆* in Figure 2 |
| DLY 4528 | W303 | *DLY 8460 nmd2::HIS3* | Lydall lab collection | *nmd2∆* in Figure S3 |
| BY 4741 *rif2∆::KANMX* | S288C | *MATa ura3 leu2 his3 rif2::KANMX* | Peter Banks, Newcastle University | Used to knockout *RIF2* in DLY 8460 |
| DLY 6884 | W303 | *DLY 8460 yku70::LEU2* | Lydall lab collection | *yku70∆* in Figure S3 |
| DLY 11741 | W303 | *DLY 8460 mre11::URA3* | Lydall lab collection | *mre11∆* in Figure S3 |
| DLY 12522 | W303 | *DLY 3001 pDL:URA3* | Lydall lab collection | *WT* in Figure 2 (lane 2) |
| DLY 12525 | W303 | *DLY 8460 pDL:TRP1* | Lydall lab collection | *WT* in Figure 2 (lanes 7–12) |
| DLY 12528 | W303 | *DLY 8460 yku70::LEU2 pDL:TRP1* | Lydall lab collection | *yku70∆* in Figure 2 |
| DLY 12531 | W303 | *DLY 8460 rif2::HIS3 pDL:TRP1* | Lydall lab collection | *rif2∆* in Figure 2 |
| DLY 12555 | W303 | *DLY 3001 rif1::KANMX* | This study | *rif1∆* clone A in Figure 3 |
| DLY 12558 | W303 | *DLY 8460 rif2::KANMX* | This study | *rif2∆* clone A in Figure 3, *rif2∆* haploid parent in Figure 4 |
| DLY 12559 | W303 | *DLY 8460 rif2::KANMX* | This study | *rif2∆* clone B in Figure 3 |
| DLY 12564 | W303 | *DLY 3001 rif1::KANMX* | This study | *rif1∆* clone B in Figure 3 |
| DLY 12568 | W303 | *DLY 3001 long VIR telomere* | This study | *WT* in Figure 5A (lane 3), Figure S3 (lanes 2, 7, 12, 17, 22, 27–29) |
| DLY 12569 | W303 | *DLY 8460 rif2::KANMX* | This study | *rif2∆* in Figure S2 (lane 3) |
| DLY 12571 | W303 | *DLY 3001 long VIR telomere* | This study | *WT* in Figure 5B (lane 2) |

**Table 2.** Cell division numbers

| **Cell origin** | **In a colony** | **In a patch** | **In liquid culture** | **Reference** |
| --- | --- | --- | --- | --- |
| Auxotrophically selected diploids | 25 (from 1 to 2.8*10^7^ cells) | 4 (from 1.4*10^7^ to 1.6*10^8^ cells) | 3 (from 5*10^7^ to 2.9*10^8^cells) | P1 diploids in Figures 2, S1 |
| All diploids from P2 and beyond | 25 (as above) | 4 (as above) | 3 (as above) | Diploids in Figure 2, S1, and S3 (except P1); |
| Haploid transformants | 26 (1^st^ G418 plate: from 1 to 4.6*10^7^ cells) and 23 (2^nd^ G418 plate: from 1 to 6.8*10^6^ cells) | 5 (from 6.8*10^6^ to 1.6*10^8^ cells) | 3 (est. from data for haploids) | P1 haploids in Figure 3 |
| Haploids from P2 and beyond | 26 (from 1 to 7.9*10^7^ cells) | 3 (from 2.0*10^7^ to 15.3*10^7^ cells) | 3 (from 5.1*10^7^ to 3.6*10^8^ cells) | Haploids in Figure 3 (except P1) |
| Pulled zygote | 25 (from 1 to 2.8*10^7^ cells) |  | 4 (from 1.4*10^7^ to 1.8*10^8^ cells) | Diploids in Figures 4, 5, and S2; P1 in Figure S3 |
| Germinated spore | 25 (from 1 to 2.4*10^7^ cells) |  | 4 (from 1.2*10^7^ to 2.1*10^8^ cells) | Haploid progeny in Figures 4, 5, and S2 |

**Table 3.** Primers used in this study

| **Primer** | **Annealing region** | **Sequence** | **Used for** | **Origin** |
| --- | --- | --- | --- | --- |
| M294 | Y’-TG | CCCAGTCACGACGTTGTAAAACG | Y’-TG probe | M13/pUC Forward |
| M295 | Y’-TG | AGCGGATAACAATTTCACACAGG | Y’-TG probe | M13/pUC Reverse |
| M1105 | TEL06R | TAAAGGAATCCCCAGAGACCTC | VIR probe | (Zubko and Lydall 2006) |
| M1106 | TEL06R | TTGCCACGCAAAGAAAGG | VIR probe | (Zubko and Lydall 2006) |
| M4813 | *RIF1* knockout (S288C) | CGCTATCAGCCAAGTATAACG | Amplifying G418^r^ cassette inserted into *RIF1* locus of S288C strain to knock out *RIF1* in W303 background | This study |
| M4814 | *RIF1* knockout (S288C) | CACAAATTAGAGTAAAACCCGACC | Amplifying G418 construct inserted into *RIF1* locus of S288C strain to knock out *RIF1* in W303 background | This study |
| M4815 | *RIF2* knockout (S288C) | GAGCGTTGGAAATGATAGCG | Amplifying G418^r^ cassette inserted into *RIF2* locus of S288C strain to knock out *RIF1* in W303 background | This study |
| M4816 | *RIF2* knockout (S288C) | GGTAAAGATCAGTCCATTCGG | Amplifying G418 construct inserted into *RIF2* locus of S288C strain to knock out *RIF1* in W303 background | This study |
| M4821 | TEL03L | AGCTTTCATCATTCGCGCTGA | IIIL probe | Raymund Wellinger, pers. comm. (2024) |
| M4822 | TEL03L | CGTCAACAGGTTATGAGCCCT | IIIL probe | Raymund Wellinger, pers. comm. (2024) |
| M4825 | TEL15L | CTTCGTCAAAGGGATGAAAGC | XVL probe | This study |
| M4826 | TEL15L | GGTAATGATGCTATGCTCTTAACG | XVL probe | This study |
